# Molecular and cytogenetic description of somatic hybrids between *Gentiana cruciata* L. and *G*. *tibetica* King

**DOI:** 10.1007/s13353-019-00530-x

**Published:** 2019-11-16

**Authors:** Karolina Tomiczak

**Affiliations:** Department of Conservation Biology of Plants, Polish Academy of Sciences Botanical Garden – Center for Biological Diversity Conservation in Powsin, Prawdziwka 2, 02-973 Warsaw, Poland

**Keywords:** AFLP, CAPS, Electrofusion, Gentian, In situ hybridization, ISSR

## Abstract

Somatic hybridization provides an opportunity to create cells with new genetic constitution. Here, the interspecific somatic hybrid plants regenerated in vitro following fusion of cell suspension–derived protoplasts of tetraploid Cross Gentian (*Gentiana cruciata* L., 2*n* = 52) with protoplasts released from mesophyll tissue of another tetraploid species, Tibetan Gentian (*G. tibetica* King, 2*n* = 52), were studied. According to the results of genome analyses with AFLP, ISSR, and CAPS markers, all somatic hybrids were genetically closer to “suspension” fusion partner *G*. *cruciata* than to “mesophyll” partner *G*. *tibetica*, but they got *G*. *tibetica* chloroplasts. Chromosome counting revealed little variation in the number of chromosomes in hybrid’s cells (2*n* = 88 or 2*n* = 90), although all plants possessed similar nuclear DNA content which remained stable even after 2 years of in vitro culture. Fluorescence in situ hybridization (FISH) showed that hybrids possessed 4 to 7 chromosomes bearing 5S rDNA sites and 6 or 7 chromosomes with 35S rDNA sites. A part of FISH signals was smaller than those observed in the parental species, which could indicate the loss of rDNA sequences. Genomic in situ hybridization (GISH) showed the predominance of the number of *G*. *cruciata* chromosomes over chromosomes of *G*. *tibetica*. However, a significant level of cross-hybridization was observed for about one-third of hybrid chromosomes, indicating a high degree of homeology between the genomes of *G*. *cruciata* and *G*. *tibetica*.

**Electronic supplementary material:**

The online version of this article (10.1007/s13353-019-00530-x) contains supplementary material, which is available to authorized users.

## Introduction

*Gentiana cruciata* L. and *Gentiana tibetica* King are two endangered tetraploid species of great importance in herbal medicine and horticulture. *Gentiana cruciata* (Cross Gentian) is widespread throughout most of Europe and in Western Asia, but it is nowhere very common (Köhlein 1991). Its underground parts (roots and rhizomes) are the source of secoiridoid glycosides such as gentiopicroside, swertiamarine, and sweroside (Szucs et al. 2002). These secondary metabolites can also be found in roots of plants propagated in vitro (Hayta et al. 2011). Besides its pharmaceutical value, Cross Gentian is also an attractive plant for large rock gardens and a host for the endangered parasitic butterfly *Phengaris rebeli* (Oškinis 2012). *Gentiana tibetica* (Tibetan Gentian) grows in western Nepal, Buthan, and south-eastern Tibet (Köhlein 1991). It is an important herbaceous plant in traditional Chinese and Tibetan medicine (Tan et al. 1998; Zhao et al. 2010) and is a source of gentiopicroside, sweroside, loganic acid, *β*-sitosterol, and daucosterol (Tan et al. 1998). The presence of secoiridoid glycosides was confirmed in callus and in regenerants of *G*. *tibetica* obtained in vitro (Skrzypczak-Pietraszek et al. 1993). Because it is easy to grow in ordinary garden soil, it is seen in quite a number of gardens (Köhlein 1991).

Somatic hybridization provides an opportunity to create cells of new genetic constitution. From a practical point of view, gentian somatic hybrids could be utilized as new ornamental cultivars or as valuable herbaceous plants with unique profiles of secondary metabolites (Wang et al. 2011). However, the merger of two nuclear genomes within a conjoint cytoplasmic environment can result in a genomic shock causing rapid and extensive alternations at the genetic and epigenetic levels (Sun et al. 2014; Liu et al. 2015; Jia et al. 2017). As a result, somatic hybrids can experience whole and/or partial chromosome elimination or recombination (Babiychuk et al. 1992; Buiteveld et al. 1998b; Wang et al. 2008; Cui et al. 2015), polyploidy (Trabelsi et al. 2005; Tomiczak et al. 2017), and organelle segregation (Sundberg and Glimelius 1991; Walters and Earle 1993; Buiteveld et al. 1998a). Also, protoplast culture itself is frequently associated with the genetic instability of regenerated plants (Tomiczak et al. 2015b; Tomiczak et al. 2016). Thus, a manifold and detailed description of the somatic hybrids obtained with the use of cytomorphological, cytogenetic, molecular, and biochemical tools is always essential.

Recently, somatic hybrid calli and plants produced by protoplast electrofusion between diploid *Gentiana kurroo* Royle and tetraploid *G*. *cruciata* have been characterized (Tomiczak et al. 2017). Greater genetic similarity of all hybrids to the species of higher ploidy (i.e., *G*. *cruciata*) and the inheritance of chloroplasts from this particular fusion partner were unveiled using AFLP, ISSR, and CAPS markers. As a consequence of polyploidization, probably occurring early in the post-fusion culture, a high degree of genetic instability, manifesting itself in a stepwise reduction of total DNA content, poor rooting, and low viability in vitro, was also observed.

Here, another group of somatic hybrids in the genus *Gentiana*, concretely plants regenerated after symmetric electrofusion of cell suspension– and leaf mesophyll–derived protoplasts of two tetraploid species, namely, *G. cruciata* and *G. tibetica* (Tomiczak et al. 2015a), is analyzed. According to previous studies and data presented in the literature, both species possess 52 (2*n* = 4*x* = 52) small and poorly identifiable metacentric and submetacentric chromosomes (Yuan et al. 1998; Tomiczak et al. 2016, 2017). To improve the cytogenetic description of *Gentiana* species and somatic hybrids, for the first time, the methods of FISH and GISH were used. This first technique supported by the use of repetitive DNA sequences like genes encoding ribosomal RNA (rDNA) as probes provides important markers for chromosome discrimination (Cuco Silvia et al. 2005), while the other is unmatched for identification of individual genomes in hybrids and allopolyploids (Garcia et al. 2017)

The study was aimed at (1) characterization of the molecular background of nuclear and chloroplast DNA composition of somatic hybrids between *G*. *cruciata* and *G*. *tibetica*, (2) identification of the number and chromosomal location of rDNA loci in *G*. *cruciata*, *G*. *tibetica*, and their somatic hybrids with the help of rDNA-FISH, and (3) determination of the parental origin of the chromosomes in somatic hybrids between *G*. *cruciata* and *G*. *tibetica* using GISH.

## Material and method

### Plant material

Experiments were carried out on interspecific somatic hybrid plants, consecutively regenerated as independent somatic embryos from a single hybrid callus line F30A (Tomiczak et al. 2015a) following electrofusion of protoplasts released from 2-year-old embryogenic cell suspension derived from cotyledons of *G*. *cruciata* L. (CR/C; Mikuła et al. 2005) and protoplasts isolated from leaf mesophyll of *G*. *tibetica* King (Tomiczak et al. 2016). Plants marked as F30A-1, F30A-2, F30A-3, and F30A-4 were obtained 38 weeks after protoplast fusion, while the rest (F30A-5, F30A-6, and F30A-7) about 6 weeks later. The reference plant material was seed-derived in vitro–grown plants of *G*. *cruciata* and *G*. *tibetica* as well as cell suspension of *G*. *cruciata*. All plants were grown in glass jars on medium composed of full MS (Murashige and Skoog 1962) mineral salts and vitamins, 30 g L^−1^ sucrose, and 8 g L^−1^ agar. Cell suspension of *G. cruciata* was maintained in a 250-mL Erlenmeyer flask filled with 80 mL liquid MS medium enriched with 30 g L^−1^ sucrose, 1.0 mg L^−1^ dicamba, 0.1 mg L^−1^ α-naphthaleneacetic acid, 2.0 mg L^−1^ 6-benzylaminopurine, and 80 mg L^−1^ adenine sulfate, on a rotary shaker at 120 rpm. Plant subcultures to new medium were set up every 5 months, while cell suspension was subcultured every week. All cultures were maintained in a phytotron at a temperature of 21 ± 1 °C and a 16-h photoperiod. Light intensity of 100 μM m^−2^ s^−1^ for plants and 20 μM m^−2^ s^−1^ for cell suspension was provided by standard daylight fluorescent tubes.

### DNA extraction

Leaves of somatic hybrids and 6 *G. tibetica* plants, as well as 6 samples of *G. cruciata* cell suspension tissue, were used for extracting total genomic DNA. The extraction, quality check, and quantitation of DNA were carried out in the same manner as previously reported (Tomiczak et al. 2017).

### Nuclear DNA analysis with AFLP and ISSR markers

Molecular analyses were performed according to Tomiczak et al. (2017) with the use of 10 selective PCR primer combinations for AFLP (Table 1) and 10 PCR primers for ISSR (Table 2). All AFLP and ISSR fragments amplified were scored and merged into binary matrices. Genetic uniformity among parental species and somatic hybrids was evaluated by calculation of the Jaccard similarity coefficient and UPGMA clustering analysis using XLSTAT Version 2016.01.26779 software. The nuclear genome composition of somatic hybrids was determined as previously described (Tomiczak et al. 2017) by counting preserved, deleted, and unique markers.

**Table 1:** AFLP primers used in selective PCR reactions

No. of primer pair	Eco/Mse primers	No. of bands	No of polymorphic bands	% polymorphism
I	E-ACG/M-CGC	20	13	65.0
II	E-AGC/M-CAC	45	25	55.6
III	E-AGG/M-CTG	44	27	61.4
IV	E-ACT/M-CCC	39	20	51.3
V	E-ATG/M-CGA	23	9	39.1
VI	E-AAA/M-CCG	39	19	48.7
VII	E-ATC/M-CAA	80	42	52.5
VIII	E-AGA/M-CAG	38	19	50.0
IX	E-ACC/M-CGT	32	22	68.8
X	E-AGT/M-CTC	70	44	62.9
	Total	430	240	55.8

**Table 2:** Sequences of primers used for ISSR analysis

Primer code	Sequence 5′− > 3′	No. of bands	No. of polymorphic bands	% polymorphism
UBC-814	(CT)_8_A	6	6	100.0
UBC-818	(CA)_8_G	15	13	86.7
UBC-835	(AG)_8_YC	5	3	60.0
UBC-840	(GA)_8_YT	17	11	64.7
UBC-846	(CA)_8_RT	9	4	44.4
UBC-880	(GGAGA)_3_	14	4	28.6
IS-2	(GAC)_4_RC	10	6	60.0
IS-811	(AC)_8_C	5	3	60.0
SBS-861	(ACC)_5_	11	7	63.6
SBS-862	(AGC)_5_	9	6	66.7
	Total	101	63	62.4

### Development of CAPS markers and analysis of cpDNA

Polymorphism in cpDNA of parental species and the transmission of chloroplasts to somatic hybrids were analyzed by PCR amplification of the *atp*B*-rbc*L region followed by its restriction digestion. Primer3 software (Rozen and Skaletsky 1999) and nucleotide sequences available in NCBI database for *atp*B*-rbc*L region of gentians were used to design appropriate primers (forward *atp*Bf: 5′-ACCAGAACCGGAAGTAGTCG-3′ and reverse *rbc*Lr: 5′-TAGCGCAACCCAATTTTTCT-3′). The PCR reaction and the amplicon sequencing were conducted as reported elsewhere (Tomiczak et al. 2017). The nucleotide sequences obtained for *G. cruciata* and *G. tibetica* (GenBank accession numbers KY566219.1 and KY566221.1, respectively) were BLASTed against each other and scanned for restriction site polymorphism with the help of NEBcutter V2.0 software (Vincze et al. 2003). Subsequently, restriction analysis of amplified *atp*B*-rbc*L region of parental species and somatic hybrids was conducted. The PCR products were digested with an appropriate endonuclease according to the manufacturer’s recommendations (New England Biolabs, Ipswich, USA), run on 1.8% agarose gel, and stained with ethidium bromide. Restriction patterns obtained for *G. cruciata* and *G. tibetica* were compared with those generated for somatic hybrids.

### Flow cytometry

Flow cytometric analyses were carried out after 6 months from plant regeneration according to Tomiczak et al. (2017) except that *Petunia hybrida* “PxPc6” (2.85 pg 2C; Marie and Brown 1993) served as an internal standard for the estimation of the nuclear DNA content of all plants. Further monitoring of the genome size of selected hybrid plants was carried out for about 2 years. Flow cytometry analyses were repeated every 8 months. The average nuclear DNA content was calculated for each of the somatic hybrids based on all measurements. Means were compared using Tukey’s test, at the 0.05 level of significance, with the help of Statistica ver. 6.0 (StatSoft Polska Sp. z o.o., Poland).

### Chromosome counting and in situ hybridization

Cytogenetic analyses were conducted on preparations of metaphase chromosomes from root-tip cells of selected in vitro–grown parental and somatic hybrid plants. Roots were pretreated with 8-hydroxyquinoline and fixed as previously reported (Tomiczak et al. 2016). Chromosome preparation followed the procedure described by Hasterok et al. (2001). Briefly, fixed roots were rinsed with 0.01 M citric acid-sodium citrate buffer (pH 4.6) for at least 20 min and digested for 35 min in an enzyme mixture consisting of 20% (v/v) pectinase (Sigma-Aldrich, St. Louis, USA), 1% (w/v) cellulase (Calbiochem, San Diego, USA), and 1% (w/v) cellulase “Onozuka RS” (Yakult Honsha Co., Ltd., Tokyo, Japan) at 37 °C. The root-tips were squashed in a drop of 45% acetic acid on microscope slides. After freezing the slides in liquid nitrogen and removing coverslips, the whole preparations were post-fixed in 3:1 ethanol:glacial acetic acid, dehydrated in absolute ethanol, and air-dried.

The dual-color rDNA-FISH was carried out on metaphase chromosomes of in vitro–grown *G*. *cruciata*, *G*. *tibetica*, and selected somatic hybrids according to Hasterok et al. (2001) with minor modification. The tandem repeat sequences, 5S rDNA (pTa794; Gerlach and Dyer 1980) labeled by PCR with rhodamine and 26S rDNA (2.3 kbp fragment of the 25S rDNA coding region of *A*. *thaliana*; Gerlach and Dyer 1980) labeled by nick-translation with digoxigenin-11-dUTP (Roche, Basel, Switzerland), were used as probes. Selected preparations of chromosomes were pretreated with RNase A (100 μg mL^−1^) in 2 × SSC for 1 h at 37 °C, rinsed twice in 2 × SSC for 5 min, then post-fixed in 1% formaldehyde in phosphate buffered saline (PBS) for 10 min, again washed twice in 2 × SSC for 5 min, dehydrated in an ethanol dilution series, and finally air-dried. The hybridization mixture comprised 50% deionized formamide, 10% dextran sulfate, 2 × SSC, 0.5% sodium dodecyl sulfate (SDS), probe DNA (75–100 ng per slide), and salmon sperm blocking DNA (10 μg per slide). Probes were pre-denatured at 75 °C for 10 min and applied onto chromosome preparations. Denaturation of preparations with probes was conducted at 70 °C for 4 min 30 s and followed by overnight hybridization at 37 °C in a moist chamber. Following hybridization, slides were rinsed twice for 5 min in 10% formamide in 0.1 × SSC at 42 °C, and twice in 2 × SSC at 42 °C and at 20 °C. Digoxygenated probes were immunodetected by FITC-conjugated anti-digoxygenin antibodies (Roche, Basel, Switzerland). The chromosome preparations were mounted and counterstained in Vectashield H-10 (Vector Laboratories, Burlingame, USA) enriched with 2.5 mg mL^−1^ 4′,6-diamidino-2-phenylindole (DAPI; Sigma-Aldrich, Saint Louis, USA).

For GISH analysis, total genomic DNA of *G. tibetica* labeled by nick-translation with digoxigenin-11-dUTP was used as a probe. Blocking DNA was isolated from *G. cruciata* and fragmented by heating at 99 °C for 45 min. Chromosome preparations were pretreated with RNase A, post-fixed, washed, and dehydrated in the same manner as described above for FISH. The hybridization mixture consisting of 55% deionized formamide, 11% dextran sulfate, 2.2 × SSC, probe DNA (90 or 180 ng per slide), and blocking DNA (9 μg per slide) was applied in an aliquot of 20 μL onto preparations and denaturated at 76 °C for 2 min. Hybridization, post-hybridization washing, and immunodetection of digoxygenated probes were carried out as for the FISH procedure. The chromosome preparations were mounted and counterstained in Vectashield H-10 supplemented with 5 μg mL^−1^ propidium iodide (PI; Sigma-Aldrich, Saint Louis, USA).

Both FISH and GISH results were visualized using a CCD camera attached to an BX51 epifluorescence microscope (Olympus, Tokyo, Japan), electronically processed, and superimposed in dedicated Olympus “Cell^B” imaging software. For both parental species, chromosome ideograms were constructed on the basis of FISH results with the use of DRAWID software version 0.26 (http://drawid.xyz/; Kirov et al. 2017).

## Results

### Composition of somatic hybrid nuclear DNA

All somatic hybrid plants possessed AFLP (Online Resource 1a) and ISSR (Online Resource 1b) bands specific for both parents, which affirmed their hybridity. The use of 10 AFLP selective primer pairs allowed amplification of a total of 430 DNA fragments (from 20 to 80 per primer pair), of which 240 (55.8 %) were polymorphic (Table 1). Electrophoretic patterns of hybrid plants contained from 397 to 406 bands, and of these, 120–126 (30.2–31.3%) were typical for *G. cruciata*, 82–86 (20.5–21.2%) – characteristic of *G. tibetica*, 190 (46.8–47.9%) – common to both parental species, and 3 or 4 – unique, absent in electropherograms of parental species (Online Resource 2a). AFLP analysis of somatic hybrids also showed that 191 to 197 amplicons were deleted from *G. cruciata*, which represented 60.3–62.1% of all bands common with *G. cruciata*, and 215 to 219 were deleted from *G. tibetica*, which comprised 71.4–72.8% of bands common with this species. Among somatic hybrids themselves, the differences were observed for 30 AFLP bands.

By using 10 ISSR primers, in total, 101 amplicons were produced (from 5 to 17 per primer pair), of which 63 (62.4%) were polymorphic (Table 2). Electrophoretic patterns of hybrid plants contained 86 to 89 bands, and of these, 27–28 (30.7–32.6%) were typical for *G. cruciata*, 18–20 (20.5–22.7%) – characteristic of *G. tibetica*, 39 (43.8–45.3%) – common to both parents, and 1–3 – unique, absent in electropherograms of parental species. Forty-one to 42 bands were recognized as deleted from *G. cruciata*, which comprised 57.7–59.2% of all bands common with *G. cruciata*. Also, 46 bands were deleted from *G. tibetica*, which represented 69.7% of bands common with this species (Online Resource 2b). Somatic hybrids differed from one another in 5 ISSR bands.

**Fig. 1:**
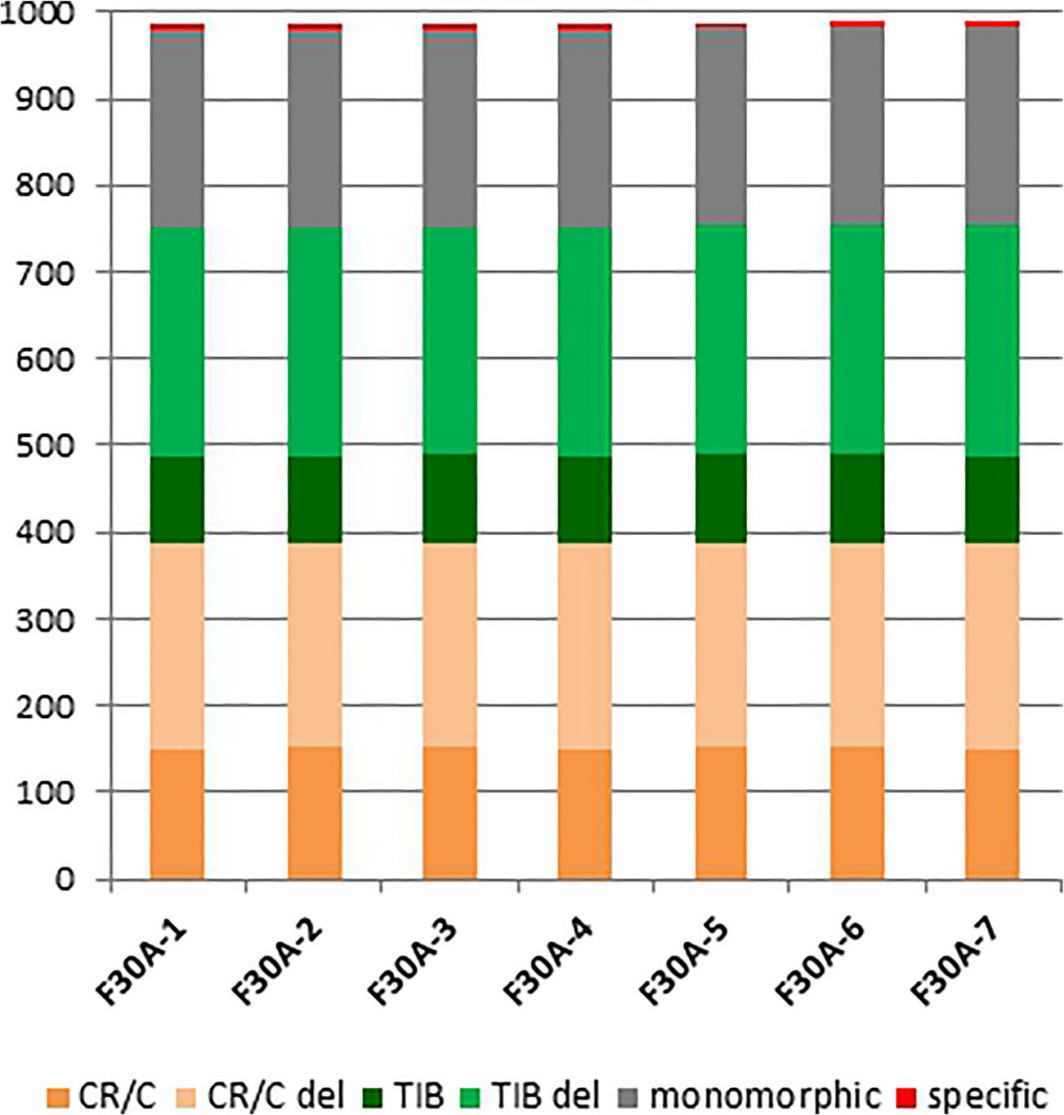
Composition of all preserved, deleted and unique AFLP and ISSR markers, detected in somatic hybrids between *G. cruciata* and *G. tibetica*. CR/C – preserved *G. cruciata* markers; CR/C del – deleted *G. cruciata* markers; TIB – preserved *G. tibetica* markers; TIB del – deleted *G. tibetica* markers

Combination of AFLP and ISSR data enabled the recognition of in total 531 bands; 485–492 of which were present in hybrids electropherograms. One hundred forty-eight to 154 bands were preserved from *G. cruciata*, which comprised 38.1–39.7% of all *G. cruciata* bands. Two hundred thirty-two to 238 (59.8–61.3%) bands common with this species were deleted. Consequently, 100–104 (27.2–28.3%) bands were inherited from *G. tibetica* and 261–265 (71.1–72.2%) were deleted (Fig. 1).

**Fig. 2:**
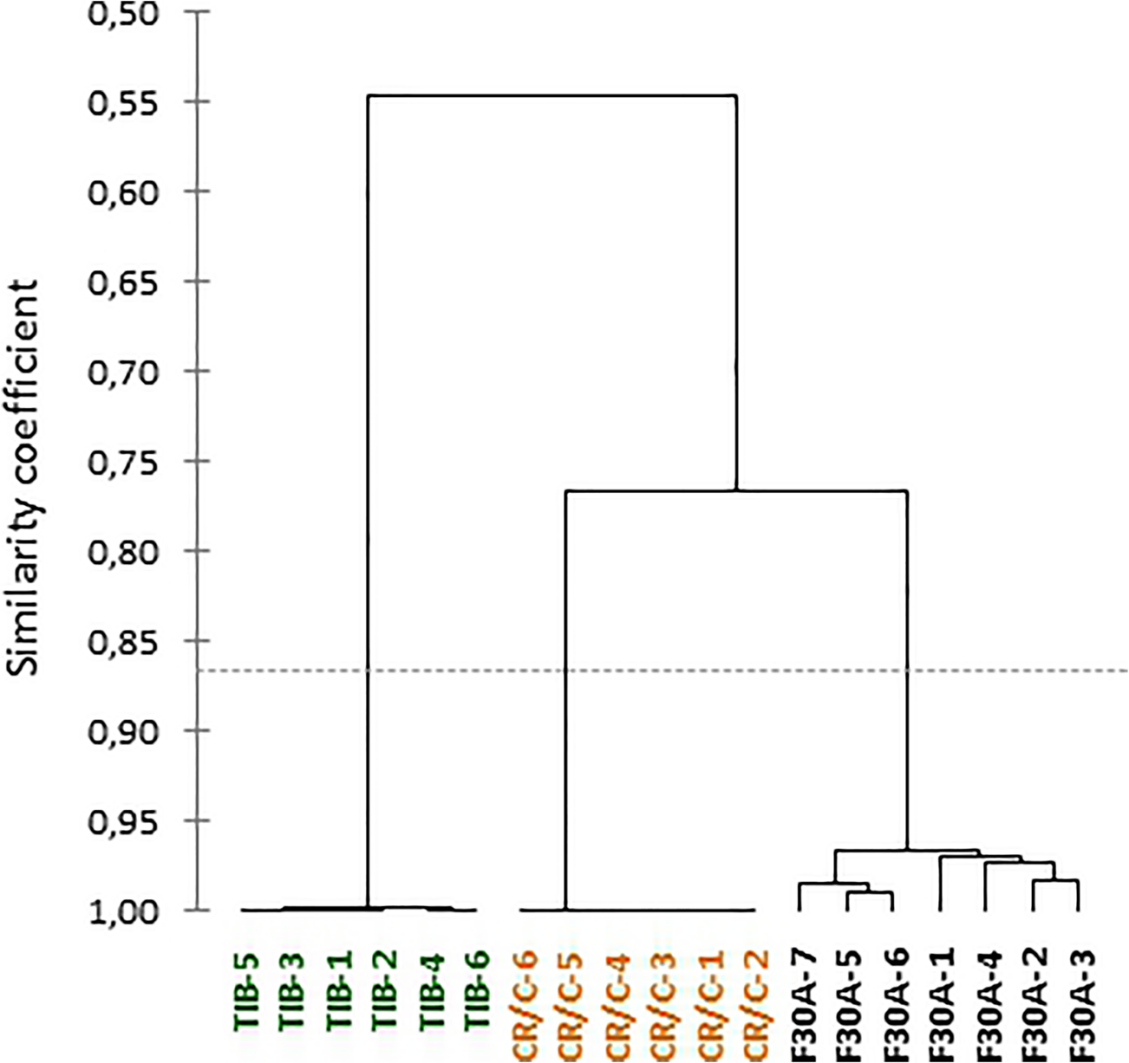
Dendrogram of genetic similarity between *G. cruciata*, *G. tibetica*, and their somatic hybrids, obtained by UPGMA cluster analysis based on combined AFLP and ISSR molecular markers. CR/C - *G. cruciata* (“cell suspension” fusion partner); TIB - *G. tibetica* (“mesophyll” fusion partner); F30A-1–7 - individual hybrid regenerants

Jaccard similarity coefficient calculated for parental species on the basis of AFLP and ISSR analyses was 0.55 and 0.51, respectively (Online Resource 3a-b). Both marker systems showed greater genetic similarity of all somatic hybrids to *G. cruciata* than to *G. tibetica*. Furthermore, although hybrids were clustered a little differently, the similarity coefficient computed for their whole group based on both marker systems was comparable and ranged from 0.96 to 0.99 for AFLP and from 0.97 to 1.0 for ISSR. An UPGMA dendrogram constructed from conjunct AFLP and ISSR data gave a similarity coefficient of 0.55 for parental species, from 0.96 to 0.99 for somatic hybrids and approximately 0.77 for somatic hybrids and *G. cruciata* (Fig. 2).

### Inheritance of cpDNA

**Fig. 3:**
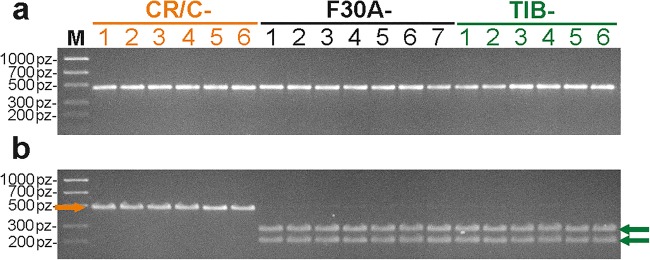
CAPS analysis of cpDNA inheritance by *G. cruciata* (+) *G. tibetica* somatic hybrids. **a** PCR amplification of an intergenic region *atp*B*-rbc*L. **b** Electrophoretic pattern of restriction digestion of PCR product with *Psi*I. CR/C - *G. cruciata* (“cell suspension” fusion partner); TIB - *G. tibetica* (“mesophyll” fusion partner); F30A-1–7 - individual hybrid regenerants; M - DNA size marker (100-bp DNA ladder). Orange arrows indicate bands specific for “cell suspension” fusion partner; green arrows indicate bands specific for “mesophyll” fusion partner

About a 470-bp fragment of *atp*B*-rbc*L region was amplified for both parental species, *G. cruciata* and *G. tibetica*, using atpBf and rbcLr primers (Fig. 3a). Amplicon sequencing and further BLAST analysis unveiled two SNPs and three insertion/deletion polymorphisms. According to the results of in silico restriction digest, one of the SNPs in a sequence of *G. tibetica* created a new restriction site for endonuclease *Psi*I. In fact, cutting of *atpB-rbcL* amplicons of *G. tibetica* with *Psi*I yielded two DNA fragments, approximately 190 and 280 bp in length, whereas the amplicons of *G. cruciata* remained uncut. The same restriction pattern as observed for *G. tibetica* was obtained for all somatic hybrid plants (Fig. 3b).

### Nuclear DNA content

Flow cytometry revealed that all somatic hybrids contained 12.19 to 12.65 pg DNA/2C after 6 months from regeneration (Table 3). This was greater than the content of parental species, i.e., 8.07 ± 0.18 pg DNA/2C for *G. cruciata* and 6.91 pg ± 0.2 pg DNA/2C for *G. tibetica* (Online Resource 4a–c), but less than the sum of the DNA content of both parents (14.98 pg). No significant reduction or increase in total DNA content of hybrids was observed following 2 years of culture. Only slight variations were observed in the total DNA content of individual regenerants and between subsequent measurements.

**Table 3:** Nuclear DNA content of selected somatic hybrids *G. cruciata* (2C = 8.07 pg) (+) *G. tibetica* (2C = 6.91 pg)

Symbol of regenerant	Nuclear DNA content (pg) after 6 months from plant regeneration	Average nuclear DNA content (pg) ± SD during 2-year-long culture
F30A-1	12.19	12.66 ± 0.38a*
F30A-2	12.43	12.92 ± 0.41*a*
F30A-3	12.43	12.68 ± 0.30*a*
F30A-4	12.54	12.70 ± 0.15*a*
F30A-5	12.54	12.69 ± 0.15*a*
F30A-6	12.65	12.76 ± 0.15*a*
F30A-7	12.65	12.74 ± 0.07*a*

*Values for a certain regenerants followed by the same letter are not significantly different at *P* = 0.05 (Tukey’s test)

### Chromosomal constitution of somatic hybrids

**Fig. 4:**
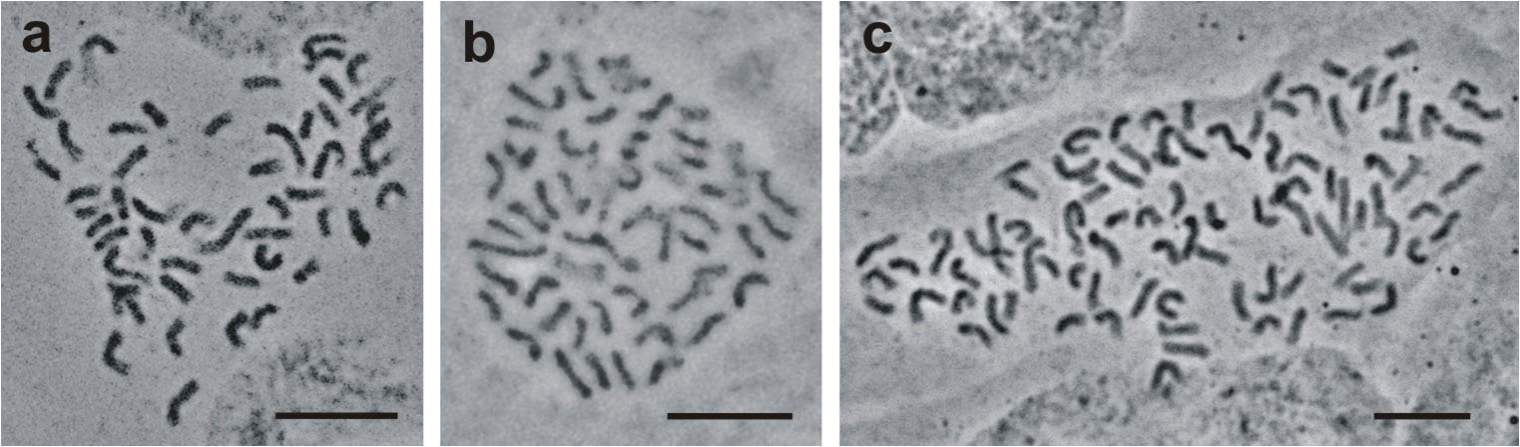
Metaphase plates of *G. cruciata*, *G. tibetica*, and somatic hybrid plant. **a** 52 mitotic metaphase chromosomes in root-tip cells of a seed-derived parent plant of *G. cruciata*. **b** 52 chromosomes in a root-tip cell of a seed-derived parent plant of *G. tibetica*. **c** 88 chromosomes in a root-tip cell of a somatic hybrid F30A-7. Scale bar = 10 μm

Cytogenetic analyses confirmed that both parental species, *G. cruciata* and *G. tibetica*, possessed 52 chromosomes in their root-tip cells (Fig. 4a–b). Of all tested metaphase plates of somatic hybrids, 79% possessed 2*n* = 88 chromosomes (Fig. 4c), whereas 2*n* = 90 chromosomes were scored for the remainder.

**Fig. 5:**
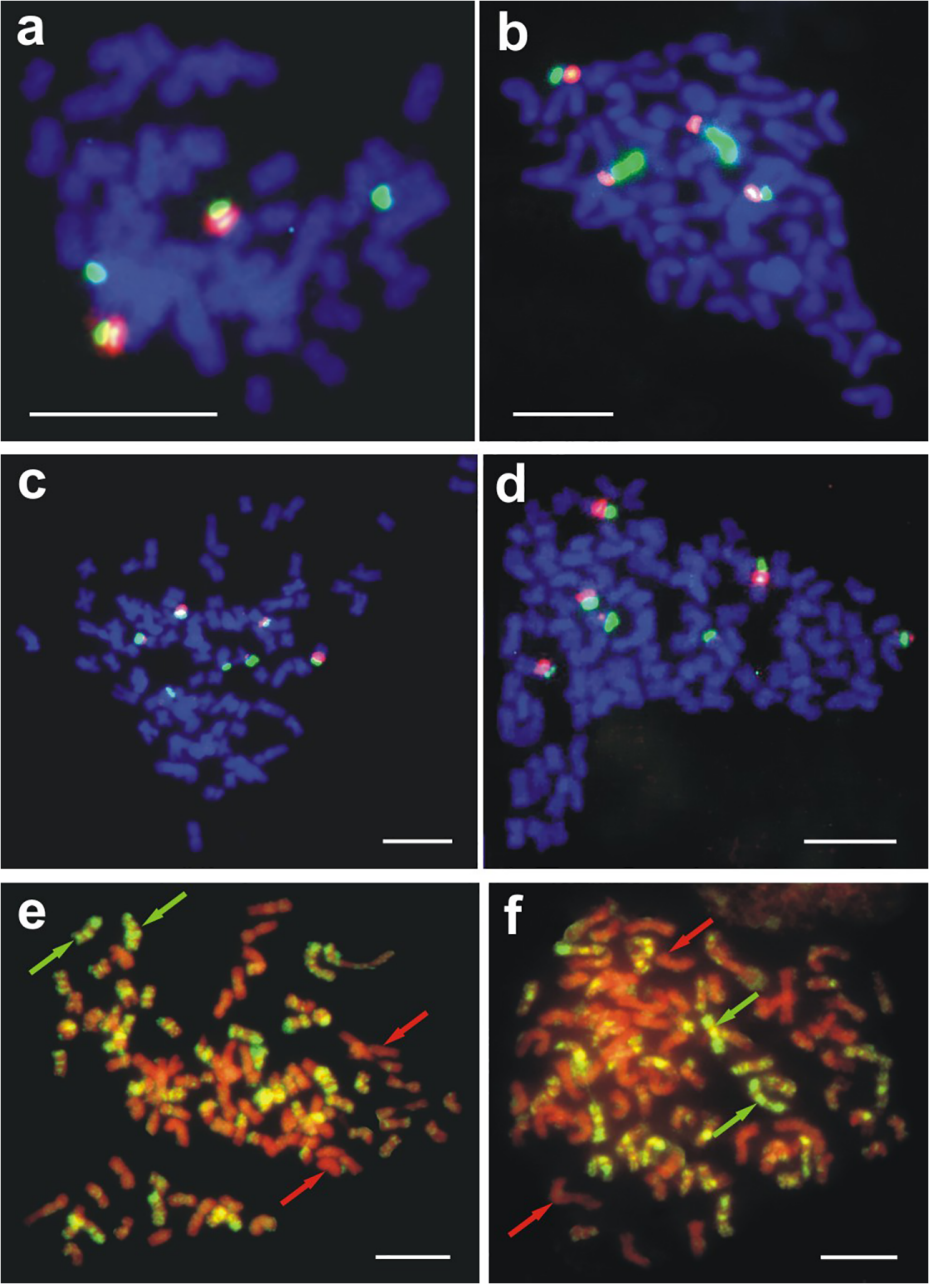
In situ hybridization analyses of root-tip metaphase chromosomes of *G. cruciata*, *G. tibetica*, and their somatic hybrids: chromosomes of *G. cruciata* after FISH showing 2 5S rDNA and 4 35S rDNA sites (**a**), chromosomes of *G. tibetica* with 4 5S rDNA and 4 35S rDNA sites (**b**), chromosomes of somatic hybrids with 5 (**c**) or 6 5S rDNA sites and 7 35S rDNA sites (**d**), chromosomes of somatic hybrids after GISH with total genomic DNA of *G. tibetica* as a probe and total genomic DNA of *G. cruciata* as a block applied at a ratio 1:100 (**e**) and 1:200 (**f**). Red and green arrows show examples of alleged chromosomes of *G. cruciata* and *G. tibetica*, respectively. Scale bar = 10 μm

**Fig. 6:**
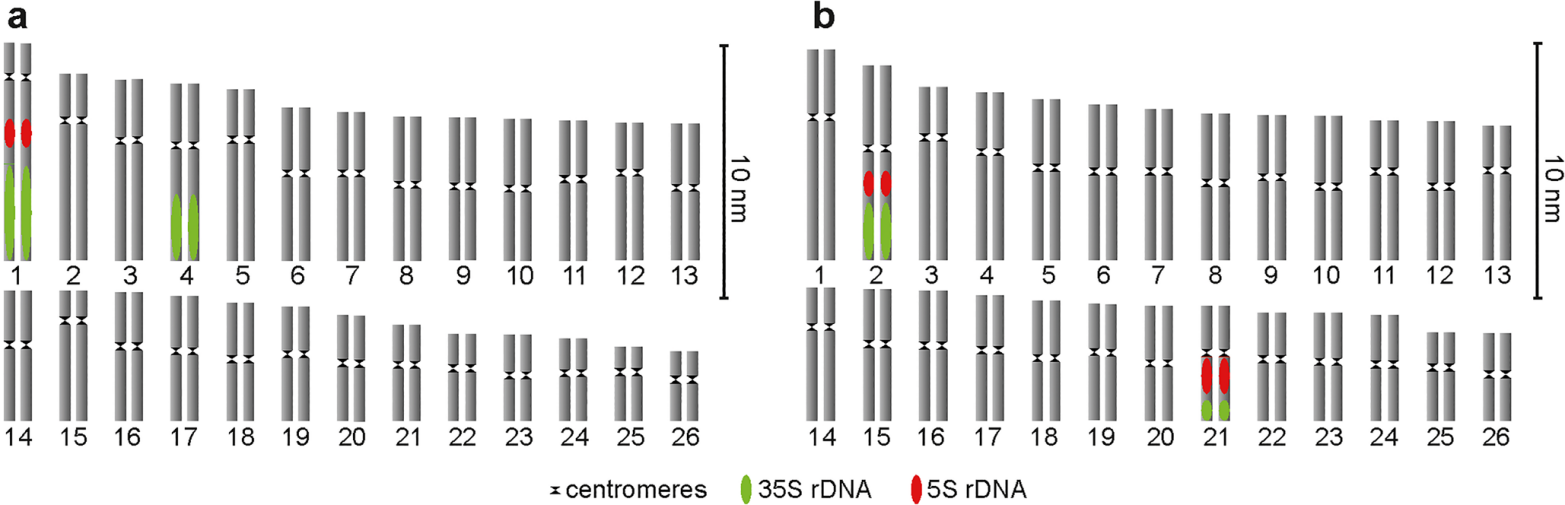
Ideograms constructed at the basis of FISH results for parental species *G. cruciata* (**a**) and G. *tibetica* (**b**)

FISH analysis allowed identifying rDNA-bearing chromosomes in all studied genomes of *Gentiana* species and somatic hybrids. One pair of chromosomes with co-localizing 5S rDNA and 35S rDNA sites, as well as one pair of chromosomes with only 35S rDNA loci, was found in the genome of *G. cruciata* by means of FISH analysis (Figs. 5a and 6a). In *G. tibetica*, two pairs of chromosomes with co-localizing 5S rDNA and 35S rDNA sites were detected (Figs. 5b and 6b). Both types of rDNA loci occupied the subtelomeric regions of the chromosomes. In somatic hybrids, FISH analysis revealed the presence of 4–7 5S rDNA-bearing chromosomes, with 5 being the most frequent (Table 4). Of all the 5S rDNA sites identified, only 3 or 4 were large and clearly visible, while the rest were smaller than those observed in parental species. All 5S rDNA loci co-localized with 35S rDNA in distal chromosome regions (Fig. 5c–d). Additional 1–3 chromosomes with only 35S rDNA sites were also found.

**Table 4:** Number of rDNA sites in four different *G*. *cruciata* (+) *G*. *tibetica* somatic hybrid plants, as revealed by FISH

Hybrid plant symbol	No. of metaphase plates tested	No. of rDNA sites (% of metaphase plates)	
		5S	35S
F30A-3	9	5 (66.7), 4 (33.3)	6 (88.9), 7 (11.1)
F30A-5	18	6 (50.0), 5 (38.9), 7 (11.1)	7 (88.9), 6 (11.1)
F30A-6	16	6 (56.25), 5 (43.75)	7 (93.75), 6 (6.25)
F30A-7	5	4 (60.0), 5 (40.0)	6 (100.0)
total	48	5 (45.8), 6 (37.5), 4 (12.5), 7 (4.2)	7 (66.7), 6 (33.3)

GISH analysis on metaphase plates of *G. cruciata* (+) *G. tibetica* somatic hybrids allowed one to distinguish between chromosomes with a strong probe hybridization signal, visible as green fluorescence, originating from the *G. tibetica* genome, and chromosomes lacking probe hybridization signals that only counterstained red, and most probably inherited from *G. cruciata* (Fig. 5e–f). Regardless of the probe:block ratio applied, the number of alleged *G. cruciata* chromosomes was greater than that for *G. tibetica* (Table 5). However, about one-third of hybrid chromosomes showed a significant level of cross-hybridization, demonstrable as a mixed red-green fluorescence.

**Table 5:** Number of chromosomes of *G. cruciata* (+) *G. tibetica* somatic hybrids showing various hybridization signals after GISH

Probe:block ratio	No. of metaphase plates tested	Mean (± SD) no. of chromosomes with hybridization signals of DNA from		
		*G. tibetica*	*G. cruciata*	Both parental species
1:100	6	21 ± 2	41 ± 2	26 ± 1
1:200	6	14 ± 0	46 ± 2	28 ± 2

## Discussion

### Molecular description of somatic hybrids

Even though, following symmetric protoplast fusion, all nuclear and organellar genetic material present in both parental species is transferred to the heterokaryon, the final genomic composition of the regenerated somatic hybrid can be very different. This is because somatic hybridization is a random genomic recombination process (Wang et al. 2008) which imitates many of the genetic modifications known to be induced by wide hybridization or polyploidization (Sun et al. 2014). However, these changes usually occur in a considerably shorter time frame and to a stronger degree than is the case in sexual hybrids (Liu et al. 2015).

Both AFLP and ISSR banding patterns of somatic hybrids between *G. cruciata* and *G. tibetica* showed that about 60% and 70% of bands were deleted from *G. cruciata* and *G. tibetica*, respectively. These fragment changes can result from a few mechanisms, among others, sequence elimination, and sequence alternations at restriction sites or at target region for primer binding (Liu et al. 2015). All somatic hybrids tested using AFLP and ISSR markers turned out to be genetically closer to *G. cruciata* (suspension parent) than to *G. tibetica* (mesophyll parent). Also, somatic hybrids between *G. kurroo* and *G. cruciata* were all closer to *G. cruciata* than to *G. kurroo* (Tomiczak et al. 2017). However, in this case, the parental species differed distinctly in terms of their ploidy, so the advantage conferred in the specific sequences of *G. cruciata* over *G. kurroo* in the genetic material of somatic hybrids could be the result of a greater degree of ploidy in *G. cruciata*. Here, *G. cruciata* and *G. tibetica* are both tetraploids with the same chromosome number. Thus, the identification of factors influencing elimination of chromosomes and/or DNA sequences needs to be further investigated.

With the use of two types of molecular markers, slight genetic differences were detected between particular somatic hybrids, too. Apart from factors related to genomic shock caused by hybridization and polyploidization, this genetic variation could also be a manifestation of somaclonal variation. Indirect regeneration via callus tissue and the increased age of culture are known to contribute to the accumulation of genetic alterations in cultured cells (Kuznetsova et al. 2006; Rathore et al. 2011; Landey et al. 2015). The separate clustering of somatic hybrids regenerated earlier (F30A-1, F30A-2, F30A-3, and F30A-4) and later after protoplast fusion (F30A-5, F30A-6, and F30A-7) may support the assumption of the effect of culture age on the nuclear DNA composition of somatic hybrids.

Besides nuclear DNA, the composition of cytoplasmic genomes is another uncertain factor in symmetric protoplast fusion, especially since somatic hybridization enables transmission, mixing, and recombination of organellar DNA (Guo et al. 2004). The inheritance of chloroplasts and mitochondria has been studied repeatedly in different combinations of species and protoplast source cells (Levi et al. 1988; Bonnema et al. 1992; Li and Sink 1992; Buiteveld et al. 1998a; Mohapatra et al. 1998; Moreira et al. 2000). In most somatic hybrids, rapid chloroplast segregation was observed, but the character of this process (random or biased), as well as the factors influencing it (including source tissue for protoplast isolation, genetic similarity of parental species, and differences in their ploidy level), has not been completely elucidated (Walters and Earle 1993). In our earlier experiments, somatic hybrids between *G. kurroo* and *G. cruciata* inherited chloroplasts from the mesophyll parent, namely, the tetraploid *G. cruciata*, whose DNA predominated in the hybrid nucleus (Tomiczak et al. 2017). This may indicate that both source cell type and nuclear genome composition might impact on chloroplast transmission to the newly created somatic hybrid. However, all somatic hybrids of *G. cruciata* (+) *G. tibetica* exhibited the same restriction pattern of cpDNA sequence as their mesophyll parent *G. tibetica*, in spite of the predominance of *G. cruciata* DNA in the nucleus and its greater DNA content than *G. tibetica*. Thus, non-random inheritance of chloroplasts seems to be determined by the parental cell type, the source of isolated protoplasts. Similar dependency was observed in *Solanum lycopersicum* L. (+) *Solanum lycopersicoides* hybrids (Li and Sink 1992).

### Cytogenetic description of somatic hybrids

According to Sun et al. (2014), genomic changes induced by genomic shock usually occur soon after the formation of hybrid cells. Either an increase or a reduction in DNA content or chromosome number has been reported for many regenerated somatic hybrids, including *Solanum brevidens* (+) *S*. *tuberosum* (Puite and Schaart 1993), *Diospyros glandulosa* (+) *D*. *kaki* (Tamura et al. 1998), and *Sinapis alba* (+) *Brassica juncea* (Kumari et al. 2018). Conversely, a gradual reduction in DNA content was observed during the process of shoot organogenesis, as in some hybrid lines of *Primula malacoides* (+) *P*. *obconica* (Mizuhiro et al. 2001), or even following plant regeneration, as was shown for somatic hybrids between *Solanum tuberosum* and *S*. *chacoense* (Guo et al. 2010) and *Gentiana kurroo* and *G. cruciata* (Tomiczak et al. 2017).

All somatic hybrids studied here contained more DNA in their nuclei than did their parental species individually, but distinctly less than anticipated after totaling the nuclear DNA content of both *G. cruciata* and *G. tibetica*. This indicates that elimination of genetic material must have occurred prior to plant regeneration. However, in contrast to the somatic hybrids *G. kurroo* (+) *G. cruciata*, no significant reduction in their total nuclear DNA content was detected after the following 2 years of culture, and plant genome size generally remained stable.

Despite possessing predominately uniform nuclear DNA content, somatic hybrids may show some variability in chromosome number. For example, hybrids between *S. lycopersicum* L. and S*. lycopersicoides*, possessing DNA content equal to the sum of that of the two parent species, had 46 to 53 chromosomes, with 51 being observed most frequently (Kulawiec et al. 2003). In hybrids of *Allium ampeloprasum* (+) *A*. *cepa*, number of chromosomes in metaphase plates of root-tip cells varied from 41 to 45, which was remarkably less than the totaled chromosome numbers of the parental species (Buiteveld et al. 1998b). Somaclones derived from four somatic hybrids between *Passiflora edulis* and *P*. *amethystina* had 36 chromosomes, but in samples of two hybrids, only 35 chromosomes were observed (Cuco Silvia et al. 2005). In root-tip cells of our somatic hybrids, 88 chromosomes were mainly present, but in 21% cells, an additional 2 chromosomes were observed.

Owing to the lack of morphological polymorphism within the majority of gentian chromosomes, it is difficult to discriminate the latter using classical cytogenetic methods. The introduction of molecular cytogenetic techniques, such as in situ hybridization, allowed us to perform more detailed studies of the genome composition of species and hybrids.

Fluorescence in situ hybridization with rDNA probes is a powerful cytogenetic tool for karyotype analysis and comparative studies of genome organizations, as well as for physical maps construction and analyzing chromosome structure and aberrations (Hasterok et al. 2001; Książczyk et al. 2010). FISH was successfully used for the exclusion of apomictic origin and verification of hybrid status in *Lilium* (Marasek et al. 2004) and for introgression analysis in hybrids of *Tulipa* (Marasek and Okazaki 2008). Cuco Silvia et al. (2005) used rDNA-FISH for karyotype analysis of three *Passiflora* species (*Passiflora amethystina*, *P. edulis* f. *flavicarpa*, and *P. cincinnata*) and for cytogenetic characterization of different *Passiflora* somatic hybrids. To the best of our knowledge, no reports exist for the use of rDNA-FISH for gentian species. Sequences of rDNA were only studied by Mel’nyk et al. (2007) in order to evaluate somaclonal variation in callus culture of *G. acaulis*, *G. punctata*, and *G. lutea*. It was shown that cultivation of gentian tissues in vitro was accompanied by a gradual reduction in the copy number of rRNA genes.

In our experiments, both 5S and 35s rDNA probes hybridized successfully with the chromosomes of either *G. cruciata* or *G. tibetica* and gave strong hybridization signals, but only on the subtelomeric regions of two pairs of chromosomes. The remaining 24 chromosome pairs were devoid of hybridization signals, and thus, still indistinguishable. Somatic hybrids possessed either chromosomes with double, red-green, hybridization signals (both 5S and 35s rDNA) or chromosomes with only 35S rDNA sites. It is likely that the former was inherited either from *G. tibetica* or *G. cruciata*, whereas the latter originated from the *G. cruciata* genome. Variability observed in the number of rDNA sites and smaller 5S rDNA signals in hybrids than in parental species suggests the partial elimination of these sequences, but without the complete loss of any of these loci or their transposition. Similar findings were reported in *Passiflora* somatic hybrids (Cuco Silvia et al. 2005).

In situ hybridization, using the genomic DNA of one or two species as a probe, is an effective way of identifying chromosomes from different sources in interspecific hybrids (Ramzan et al. 2017). It has often been used for characterizing somatic hybrids, especially from the families Brassicaceae (Tu et al. 2008; Du et al. 2009), Poaceae (Cai et al. 2007), Solanaceae (Escalante et al. 1998; Kulawiec et al. 2003), or Amaryllidaceae (Buiteveld et al. 1998b; Yamashita et al. 2002). However, in the family Gentianaceae, GISH has been used only by Wang et al. (2011), who reported elimination of most of *Swertia mussotii* chromosomes and recombination of 1–3 parental chromosomes in asymmetric somatic hybrids between *S*. *mussotii* and *Bupleurum scorzonerifolium*. Unfortunately, in the case of the somatic hybrids *G. cruciata* (+) *G. tibetica*, about one-third of hybrid chromosomes showed a significant level of cross-hybridization, reflecting the high degree of *G. cruciata* and *G. tibetica* genome homeology and making the assignment of these chromosomes to particular parental genomes impossible. Even so, the predominance of *G. cruciata* chromosome number over that of *G. tibetica* in both probe:block ratios is in accordance with data obtained using DNA molecular markers.

## Conclusion

Several molecular and cytogenetic methods were used to characterize interspecific somatic hybrids produced via electrofusion of cell suspension protoplasts of *G. cruciata* with leaf mesophyll protoplasts of *G. tibetica*. Slight asymmetry, with a predominance of nuclear DNA of *G. cruciata*, and the presence of chloroplasts derived from *G. tibetica* were detected following molecular analysis. Little variations in the DNA sequence, number of chromosomes, and rDNA sites were detected, but generally, a stable nuclear DNA content was maintained by hybrids. Evaluation of the pharmaceutical value of somatic hybrids and a study of their secondary metabolite production are currently in progress.

## Electronic supplementary material

Online Resource 1 (PDF 225 kb)

Online Resource 2 (PDF 199 kb)

Online Resource 3 (PDF 239 kb)

Online Resource 4 (PDF 182 kb)

## Abbreviations

AFLP: Amplified fragment length polymorphism
CAPS: Cleaved amplified polymorphic sequence
cpDNA: Chloroplast DNA
FISH: Fluorescent in situ hybridization
GISH: Genomic in situ hybridization
ISSR: Inter-simple sequence repeat
SNP: Single nucleotide polymorphism
SSC: Saline-sodium citrate buffer
rDNA: Ribosomal DNA
