# Supplementary material for: Molecular and cytogenetic description of somatic hybrids between Gentiana cruciata L. and G. tibetica King
Source: J Appl Genet. 2019 Nov 16;61(1):13–24. doi: 10.1007/s13353-019-00530-x (PMC6968988; doi:10.1007/s13353-019-00530-x)

**Online Resource 1** Electrophoretic patterns obtained for *G. cruciata*, G. *tibetica* and their somatic hybrids: (a) with the use of AFLP primer pair IX (E-ACC / M-CGT), (b) with the use of ISSR primer UBC-818. Abbreviations: CR/C - *G. cruciata* ("cell suspension" fusion partner); TIB - *G. tibetica* ("mesophyll" fusion partner); F30A-1–7 - individual hybrid regenerants; M – DNA size marker (100-bp DNA ladder). Orange arrows indicate bands specific for "cell suspension" fusion partner; green arrows indicate bands specific for "mesophyll" fusion partner; red arrowheads indicate bands unique for somatic hybrids.

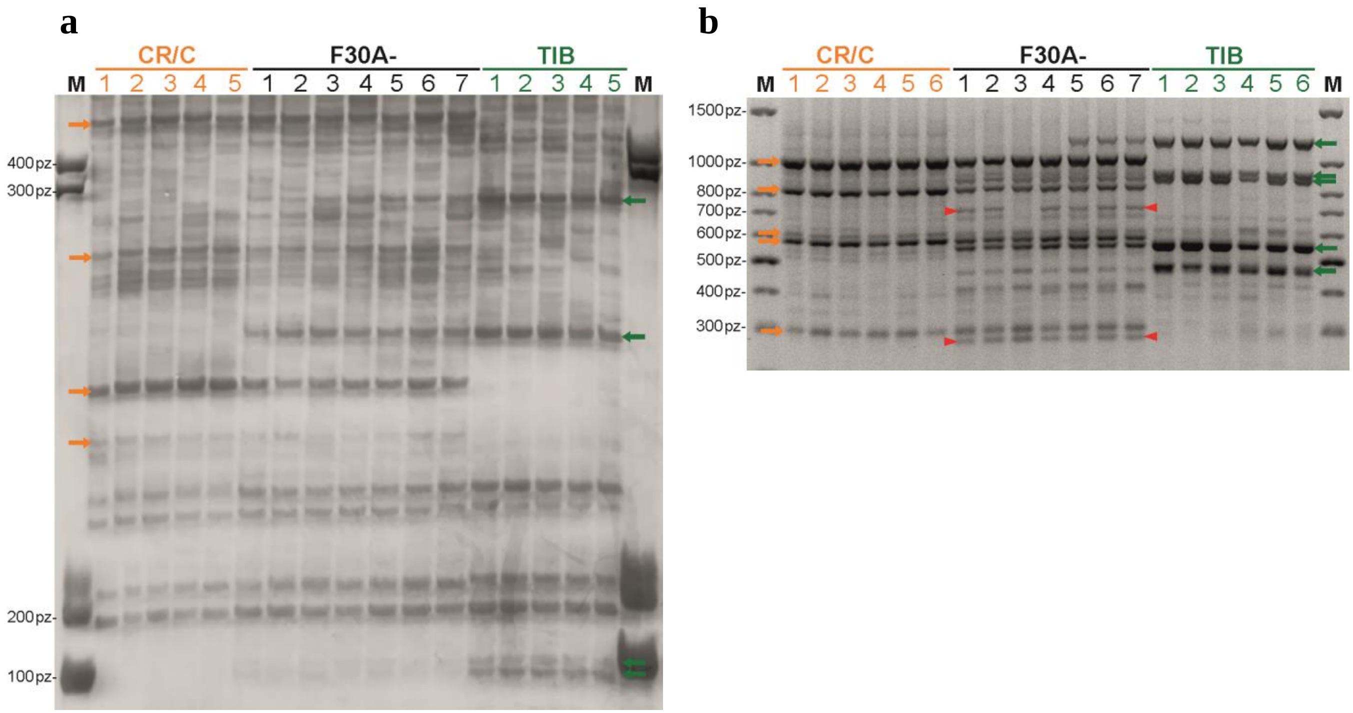

Supplement: Supplementary file 1 — (PDF 225 kb) [file 13353_2019_530_MOESM1_ESM.pdf]
