# Supplementary material for: Molecular and cytogenetic description of somatic hybrids between Gentiana cruciata L. and G. tibetica King
Source: J Appl Genet. 2019 Nov 16;61(1):13–24. doi: 10.1007/s13353-019-00530-x (PMC6968988; doi:10.1007/s13353-019-00530-x)

Online Resource 2 Composition of preserved, deleted and unique AFLP (a) and ISSR (b) markers, detected in somatic hybrids between *G. cruciata* and *G. tibetica*. Abbreviations: CR/C – preserved *G. cruciata* markers; CR/C del – deleted *G. cruciata* markers; TIB – preserved *G. tibetica* markers; TIB del – deleted *G. tibetica* markers

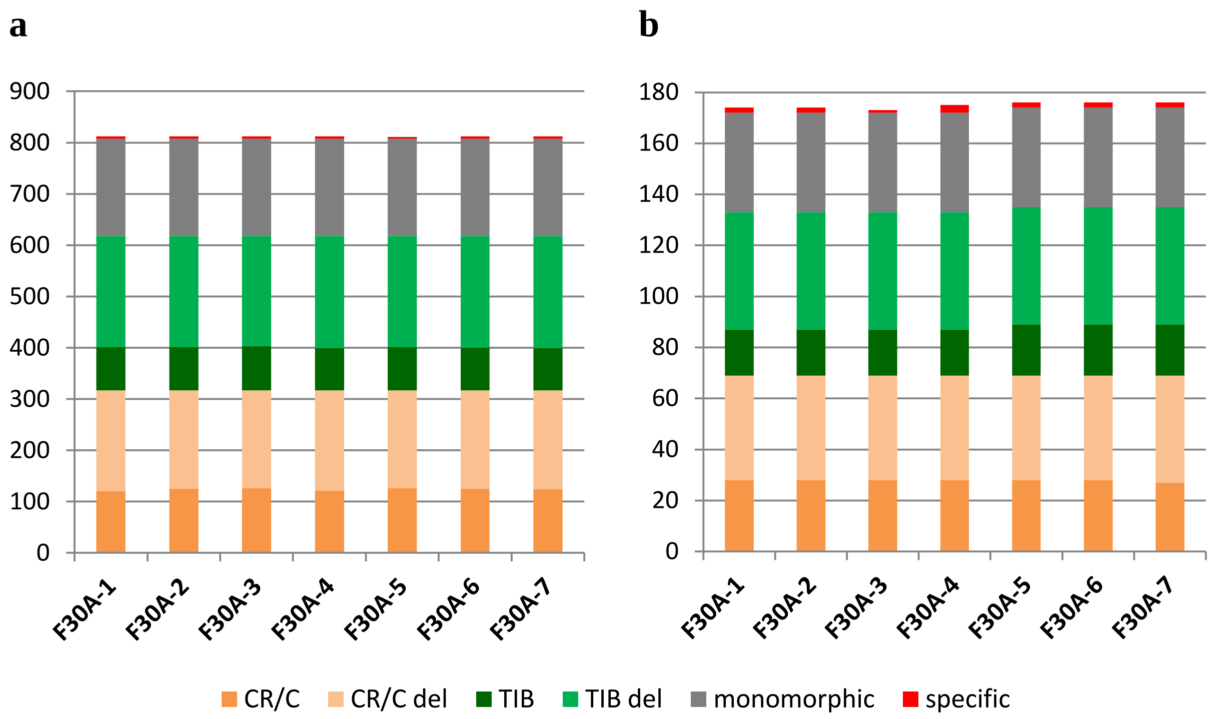

Supplement: Supplementary file 2 — (PDF 199 kb) [file 13353_2019_530_MOESM2_ESM.pdf]
