# Supplementary material for: Molecular and cytogenetic description of somatic hybrids between Gentiana cruciata L. and G. tibetica King
Source: J Appl Genet. 2019 Nov 16;61(1):13–24. doi: 10.1007/s13353-019-00530-x (PMC6968988; doi:10.1007/s13353-019-00530-x)

Online Resource 3 Dendrograms of genetic similarity between *G. cruciata*, *G. tibetica* and their somatic hybrids, obtained by UPGMA cluster analysis based on AFLP (a) and ISSR (b) molecular markers. Abbreviations: CR/C - *G. cruciata* ("cell suspension" fusion partner); TIB - *G. tibetica* ("mesophyll" fusion partner); F30A – somatic hybrid plants

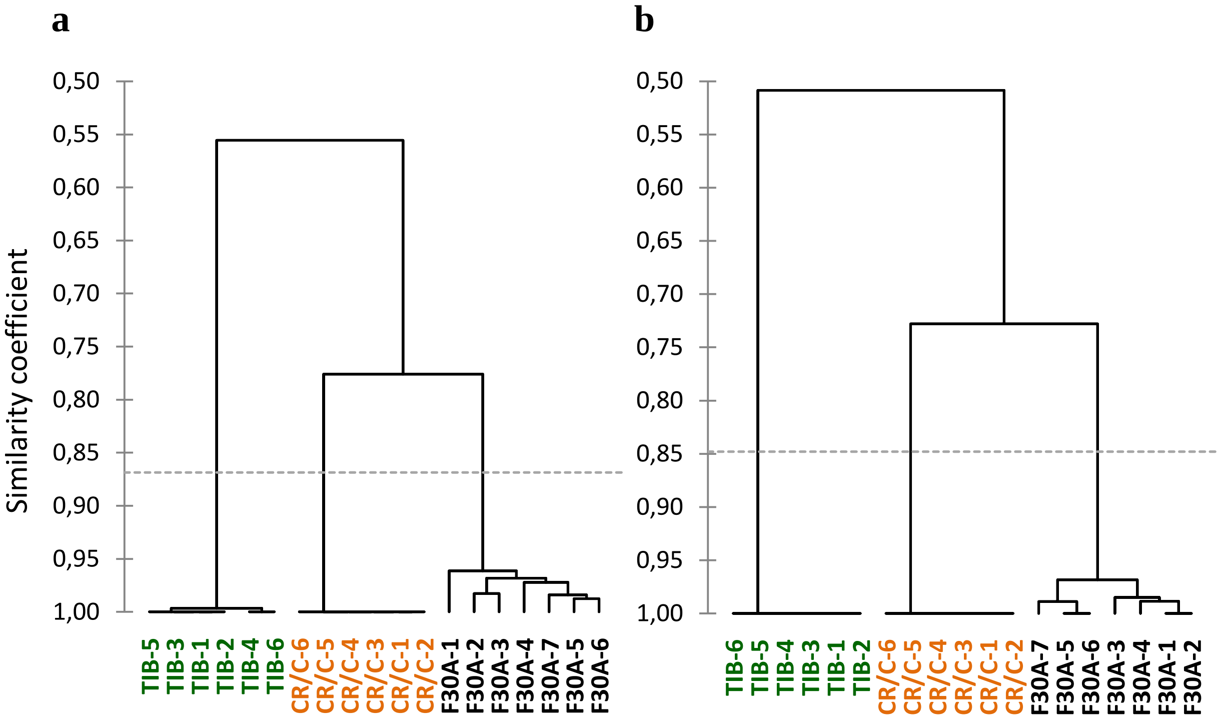

Supplement: Supplementary file 3 — (PDF 239 kb) [file 13353_2019_530_MOESM3_ESM.pdf]
