# Supplementary material for: Molecular and cytogenetic description of somatic hybrids between Gentiana cruciata L. and G. tibetica King
Source: J Appl Genet. 2019 Nov 16;61(1):13–24. doi: 10.1007/s13353-019-00530-x (PMC6968988; doi:10.1007/s13353-019-00530-x)

Online Resource 4 DNA histograms of nuclei isolated simultaneously from leaves of *P. hybrida* (internal standard) and *G. cruciata* (a), *G. tibetica* (b), somatic hybrid F30A-3 (c). Abbreviations: CR - *G. cruciata* (“cell suspension” fusion partner); TIB - *G. tibetica* (“mesophyll” fusion partner); F30A-3 – somatic hybrid plant

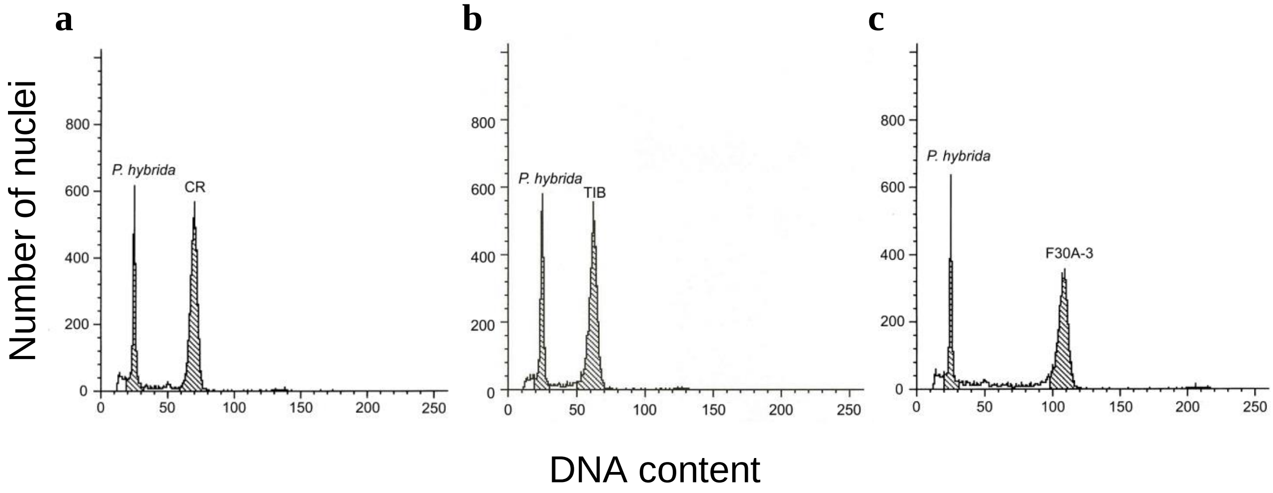

Supplement: Supplementary file 4 — (PDF 182 kb) [file 13353_2019_530_MOESM4_ESM.pdf]
